# Assessing health status and quality of life of Q-fever patients: the Nijmegen Clinical Screening Instrument versus the Short Form 36

**DOI:** 10.1186/1477-7525-11-112

**Published:** 2013-07-04

**Authors:** Joris AF van Loenhout, W John Paget, Gerwin W Sandker, Jeannine LA Hautvast, Koos van der Velden, Jan H Vercoulen

**Affiliations:** 1 Department of Primary and Community Care, Academic Collaborative Centre AMPHI, Radboud University Nijmegen Medical Centre, P.O. Box 9101, 6500 HB, Nijmegen, the Netherlands; 2 Netherlands Institute for Health Services Research (NIVEL), Utrecht, the Netherlands; 3 Department of Medical Psychology and Department of Pulmonary Diseases, Radboud University Nijmegen Medical Centre, Nijmegen, the Netherlands

**Keywords:** Q-fever, Coxiella burnetii, Health status, Quality of life

## Abstract

**Background:**

The aim of the study was to assess the use of the Nijmegen Clinical Screening Instrument (NCSI) and Short Form 36 (SF-36) in providing a detailed assessment of health status of Q-fever patients and to evaluate which subdomains within the NCSI and SF-36 measure unique aspects of health status.

**Findings:**

Patients received a study questionnaire, which contained the NCSI and SF-36. Pearson correlation coefficients between subdomains of the instruments were calculated. The response rate was 94% (309 out of 330 eligible patients). Intercorrelations between subdomains of the NCSI were generally lower than of the SF-36. Four subdomains of the NCSI showed conceptual similarity (Pearson’s r ≥ .70) with one or more subdomains of the SF-36 and vice versa. Subdomains that showed no conceptual similarity were NCSI Subjective Pulmonary Symptoms, Subjective Impairment, Dyspnoea Emotions and Satisfaction Relations, and SF-36 Social functioning, Bodily Pain, Role Physical and Role Emotional.

**Conclusions:**

Our results show that either the NCSI or SF-36 can be used to measure health status in Q-fever patients. When the aim is to obtain a detailed overview of the patients’ health, a combination of the two instruments, consisting of the complete NCSI and the four unique subdomains of the SF-36, is preferred.

## Findings

### Introduction

Q-fever is a zoonotic illness caused by the intracellular bacterium *Coxiella burnetii*. The disease was relatively uncommon in the Netherlands until 2007 and only known as an occupational illness, mainly affecting farmers, veterinarians and laboratory workers. Between 2007 and 2011, the Netherlands experienced the largest Q-fever outbreak ever reported (a total of 4108 acute notified Q-fever patients) [1]. Approximately 40% of all persons infected with Q-fever develop symptoms such as fever, pneumonia and hepatitis [2,3], but there remains a poor description of disease progression. We have established a detailed prospective cohort study to assesses the short- and long-term clinical progression of Q-fever over time [4].

Several studies have shown that a relatively large group of patients suffer from persistent fatigue and long-lasting symptoms after acute Q-fever [5-11]. Of seven published studies, two in the Netherlands used the Nijmegen Clinical Screening Instrument (NCSI) [10,11], one in Canada used the Short Form 36 (SF-36) [5] and the remaining studies used other instruments to measure health status of Q-fever patients. In the Netherlands, studies that focused on the long-term effects on health status have shown that around 50% of patients suffered from severe fatigue at least one year after onset of illness [10,11].

Patients with Q-fever experience not only severe fatigue but also many other health problems, such as dyspnoea, anxiety and frustration with dyspnoea, functional impairments in daily life, and an impaired quality of life [10,11]. This means that in order to understand the apparent complexity of the health status problems of patients with Q-fever, an instrument that provides a detailed picture of many different aspects of patients’ health status is required.

There are eight subdomains in both the NCSI and SF-36 and these measure eight aspects of health status (Table 1) [12,13]. Until now it has never been investigated whether both instruments measure the same aspects of health status or whether they complement each other when assessing the health impact of Q-fever on patients. The main aim of our study was to identify an instrument - or a combination of instruments - that would provide a detailed assessment of health status of Q-fever patients. We assessed whether subdomains within each instrument measure unique aspects of health status, or whether there is conceptual similarity between subdomains of the NCSI and SF-36. This was done by calculating intercorrelations (overlap within instruments) as well as correlations (overlap between instruments). The results will yield important information for future studies on how to measure health status of Q-fever patients.

**Table 1:** Domains, subdomains and number of questions for both the NCSI and SF-36

**Instrument**	**Domain**	**Subdomain**	**Number of questions**
NCSI	Symptoms	Subjective pulmonary symptoms	2
*Health status*		Dyspnoea emotions	6
		Fatigue	8
	Functional impairment	Behavioural impairment	22
		Subjective impairment	4
	Quality of life	General quality of life	12
		Health-related quality of life	2
		Satisfaction relations	2
SF-36	Physical health	Physical functioning	10
*Quality of Life*		Role physical	4
		Bodily pain	2
		General health	5
	Mental health	Vitality	4
		Social functioning	2
		Role emotional	3
		Mental health	5

### Methods

Patients diagnosed with Q-fever in 2010 and 2011 who were at least 18 years of age and fulfilled the Dutch notification criteria for Q-fever were eligible for this study [14]. Patients received a consent form and the study questionnaire by postal mail and those who did not respond received a reminder by telephone or postal mail. The study questionnaire consisted of the NCSI and SF-36, but it also collected socio-demographic and clinical information. The current study uses patients’ information at 12 months after onset of illness [4].

The NCSI was originally developed to provide a detailed assessment of health status of COPD patients and has been available since 2009 in English, German and Dutch. It combines a number of existing health status questionnaires [12]. The SF-36 was developed in 1988, aims to assess the quality of life of patients and is available in over 170 languages [13].

The relationships between the subdomains of the NCSI and SF-36 were calculated using Pearson correlation coefficients. A Pearson’s r ≥ .70 was used as criterion for conceptual similarity [15]. A Pearson’s r < .30 indicates no meaningful correlation.

### Results

Questionnaires were returned by 309 out of 330 patients (response rate 94%). The mean age of the study group was 49.9 (SD 13.8) and 53.7% was male. There was a statistically significant difference in age between responders and non-responders (49.9 vs. 43.1 respectively); other characteristics (gender, nationality, educational level, BMI, smoking behaviour, alcohol consumption) were not significantly different (data not shown).

Intercorrelations between the subdomains of the NCSI and SF-36 are presented in Table 2. In general, the NCSI shows lower intercorrelations between the subdomains than the SF-36. The proportion of intercorrelations ≤ .60 was 79% for the NCSI and 36% for the SF-36. Intercorrelations ≥ .70, indicating conceptual similarity, are found four times for the NCSI and three times for SF-36. In the NCSI, conceptual similarity is found between the subdomains Subjective Pulmonary Symptoms, Subjective Impairment and Dyspnoea Emotions, and between Fatigue and Health-Related Quality of Life. In the SF-36, conceptual similarity is found between Vitality on the one hand and General Health, Mental Health and Social Functioning on the other hand.

**Table 2:** Intercorrelations between the subdomains of the NCSI and SF-36

**NCSI**	**Subjective pulmonary symptoms**	**Dyspnoea emotions**	**Fatigue**	**Behavioural impairment**	**Subjective impairment**	**General quality of life**	**Health-related quality of life**	**Satisfaction relations**
Subjective pulmonary symptoms	1	*.70*	.53	.49	*.81*	.36	.47	.38
Dyspnoea emotions		1	.50	.45	*.70*	.48	.50	.37
Fatigue			1	.53	.57	.50	*.74*	.42
Behavioural impairment				1	.59	.37	.57	.40
Subjective impairment					1	.45	.59	.42
General quality of life						1	.69	.61
Health-related quality of life							1	.53
Satisfaction relations								1
**SF-36**	**Physical functioning**	**Role physical**	**Bodily pain**	**General health**	**Vitality**	**Social functioning**	**Role emotional**	**Mental health**
Physical functioning	1	.65	.63	.64	.63	.63	.48	.47
Role physical		1	.58	.62	.69	.66	.60	.50
Bodily pain			1	.61	.61	.58	.39	.42
General health				1	*.76*	.63	.46	.55
Vitality					1	*.75*	.54	*.72*
Social functioning						1	.67	.69
Role emotional							1	.66
Mental health								1

Pearson’s intercorrelations for the Nijmegen Clinical Screening Instrument (NCSI) and Short Form 36 (SF-36) for Q-fever patients at 12 months after onset of illness. N = 309^1^, p < .01 for all intercorrelations. Intercorrelations ≥ .70 are in italics.

1) N varies between 302 and 309 for individual intercorrelations, due to missing values within some of the questionnaires.

Correlations between the subdomains of the NCSI versus the SF-36 are presented in Table 3. All correlations were statistically significant (p < .01). NCSI Fatigue reached the criterion for conceptual similarity with SF-36 Vitality and General Health. NCSI Behavioural Impairment is shown to be conceptually similar to SF-36 Physical Functioning. NCSI General Quality of Life is conceptually similar to SF-36 Mental Health, and NCSI Health-Related Quality of Life is conceptually similar to SF-36 General Health and Vitality.

**Table 3:** Correlations between the subdomains of the NCSI and SF-36

**NCSI**	**SF-36**	**Physical functioning**	**Role physical**	**Bodily pain**	**General health**	**Vitality**	**Social functioning**	**Role emotional**	**Mental health**
Subjective pulmonary symptoms		.53	.43	.46	.53	.50	.51	.46	.40
Dyspnoea emotions		.50	.51	.46	.52	.50	.57	.53	.52
Fatigue		.59	.68	.61	*.72*	*.85*	.63	.47	.54
Behavioural impairment		*.71*	.60	.51	.54	.58	.58	.44	.40
Subjective impairment		.63	.54	.53	.59	.58	.59	.48	.45
General quality of life		.45	.49	.43	.53	.63	.59	.52	*.76*
Health-related quality of life		.61	.64	.58	*.71*	*.77*	.66	.54	.66
Satisfaction relations		.40	.43	.36	.46	.56	.58	.49	.60

Pearson’s correlations between subdomains of the Nijmegen Clinical Screening Instrument (NCSI) and Short Form 36 (SF-36) for Q-fever patients at 12 months after onset of illness. N = 309^1^, p < .01 for all correlations. Correlations ≥ .70 are in italics.

1) N varies between 302 and 309 for individual correlations, due to missing values within some of the questionnaires.

### Discussion

The main aim of our study was to identify an instrument or a combination of instruments that would provide a detailed assessment of health status of patients with Q-fever. We investigated whether there is conceptual similarity between the subdomains of the instruments NCSI and SF-36 or whether both instruments are complementary to each other.

The NCSI shows lower intercorrelations between the subdomains than the SF-36, indicating that the NCSI subdomains represent independent concepts to a higher degree. Some intercorrelations were very high where we did not expect this, such as between SF-36 Vitality (measuring fatigue, as indicated by the very high correlation with NCSI Fatigue) and SF-36 General Health, Mental Health and Social Functioning, which are clearly different concepts than fatigue. Similarly, NCSI Fatigue showed conceptual similarity with NCSI Health-Related Quality of Life. A possible explanation for these high intercorrelations is that fatigue is the most dominant symptom of Q-fever patients and dominates the wellbeing of the patients as expressed by other subdomains. Previous studies have already shown that Fatigue and General Quality of Life are the NCSI subdomains most severely affected in Q-fever patients at 12 months after onset of illness [10,11].

Several subdomains measure concepts of health status not measured by the other instrument. These are: NCSI Subjective Pulmonary Symptoms, Subjective Impairment, Dyspnoea Emotions and Satisfaction Relations; SF-36 Social functioning, Bodily Pain, Role Physical and Role Emotional. This means that if one wants to assess these specific subdomains, one needs to use either the NCSI or SF-36.

Conceptual similarity was found between four subdomains of each instrument, which implies that these subdomains of both instruments measure the same aspects of health status. The highest correlations were found between SF-36 Vitality and NCSI Fatigue and Health-Related Quality of Life (Pearson’s r of .84 and .77 respectively). There is conceptual similarity between NCSI Fatigue and both SF-36 General Health and Vitality, and between NCSI General Quality of Life and SF-36 Mental Health. This implies that both the NCSI and SF-36 can be used to evaluate the aspects of health status that are the most affected in Q-fever patients [10,11]. Advantages of the SF-36 are its length (only 36 questions compared to 56 in the NCSI) and the fact that it is publically available in more than 170 languages, due to which it is more often used in international studies, which makes it possible to compare the health impact of Q-fever to other diseases. The results also show that the Dutch studies which used the NCSI can be compared to international studies that used SF-36 in Q-fever patients [5]. Advantages of the NCSI are that the concepts of health status it measures are more unique and it allows a description of health status on the level of the individual (as a normal, mild or severe score is available for each subdomain).

### Conclusions

When measuring the general health status of Q-fever patients, our study shows that either the NCSI or the SF-36 can be used. Only if the aim is to obtain a detailed overview of patients’ health status, we advise that both the NCSI and SF-36, or at minimum a combination of the two, should be used. When combining the instruments we recommend that the NCSI is used (since it measures more variation in aspects of health status and because of the normative data) and it is complemented with the SF-36 subdomains not covered by the NCSI (i.e. SF-36 Role Physical, Bodily Pain, Social Functioning, Role Emotional). When the aim is to formulate a patient-centered intervention, the NCSI is preferred as it covers the important health status subdomains and since it provides a description of clinically relevant scores (normal, mild, severe) on an individual level.

## Competing interests

The authors declare that they have no competing interests.

## Authors’ contributions

JAFvL participated in the design of the study, collected data, carried out the analyses and drafted the manuscript. WJP, JLAH and JV participated in the design of the study and helped draft the manuscript. GS collected data, participated in analyses and revised the manuscript. KvdV participated in the design of the study and revised the manuscript. All authors read and approved the final manuscript.
